# Preliminary speech recognition results after cochlear implantation in patients with unilateral hearing loss: a case series

**DOI:** 10.1186/1752-1947-5-343

**Published:** 2011-08-02

**Authors:** Yvonne Stelzig, Roland Jacob, Joachim Mueller

**Affiliations:** 1 Department of Oto-Rhino-Laryngology, Central Army Hospital Koblenz, Ruebenacher Str. 170, 56072 Koblenz, Germany; 2 Department of Oto-Rhino-Laryngology, Plastic, Aesthetic and Reconstructive Head and Neck Surgery, University of Wuerzburg, Josef-Schneider-Str. 11, 97080 Wuerzburg, Germany

## Abstract

**Introduction:**

Cochlear implants known to provide support in individuals with bilateral hearing loss may also be of great benefit for individuals with unilateral hearing loss. This case report demonstrates the positive effects of cochlear implantation on speech understanding in noise conditions in patients with unilateral hearing loss and normal hearing on the contralateral side. To the best of our knowledge, the data presented here are from the first few cases to receive a cochlear implant for unilateral hearing loss.

**Case presentation:**

Four Caucasian German men, two aged 48 and the others aged 51 and 57 years old, with post-lingual unilateral hearing loss and normal hearing on the contralateral side were implanted with a cochlear implant. All our patients were members of the German army. Before and after implantation, they were given a battery of speech tests in different hearing conditions to assess the effect of unilateral cochlear implantation on speech understanding in noise conditions. Test results showed that all patients benefited from unilateral cochlear implantation, particularly in terms of speech understanding in noise conditions.

**Conclusions:**

Unilateral cochlear implantation might be a successful treatment method for patients with unilateral hearing loss not benefiting from alternative treatment options. The results of this case report open up the field of cochlear implantation for expanded criteria and new areas of research.

## Introduction

Many individuals with unilateral hearing loss (UHL) have genuine difficulties in understanding speech in noise conditions. Despite these impediments, the impact of a complete UHL is often minimized by the presence of (near) normal hearing (NH) on the contralateral side. However, different studies have shown that the normal hearing capabilities of these individuals do not compensate for their UHL [1]. Lin *et al. *[2] addressed the auditory deficits of patients with UHL. They reported that monaural patients had the greatest difficulties when the sound or source of speech was localized on the hearing impaired side, presumably due to the reduced exploitation of binaural processes.

Although it is recognized that patients with UHL encounter problems in speech recognition in noise conditions or sound localization, only very few treatment methods are available to these patients [1]. Currently, they are generally treated with contralateral routing of signals (CROS) hearing aids [3] or bone-anchored hearing aid (BAHA) implants [4]. However, various studies have demonstrated a poor user satisfaction of CROS as well as only a minimal improvement in speech discrimination in noise conditions and none for sound localization with the BAHA implant [2,5].

In contrast to these treatment options for UHL, bilateral HL is mainly treated by cochlear implantation. The effectiveness of bilateral cochlear implants (CIs) has been demonstrated in the last several years [6,7]. Patients with bilateral CIs show better speech discrimination largely due to the exploitation of the head shadow effect (with the head obstructing noise sources from the receiving ear), the binaural summation effect (receiving redundant information at both ears) and the squelch effect (occurring due to temporal and spectral differences of spatially separated speech and noise sources) [8]. According to Schleich *et al. *[9], bilateral CI users significantly benefit from head shadow, squelch and summation effects, substantially improving their performance of speech understanding in noise conditions. These results may also suggest that individuals with UHL could gain substantial benefit from CIs due to added binaural effects as shown in a tinnitus study by Vermeire *et al. *[10].

## Case presentation

Patient S1 was a 48-year-old Caucasian German man with post-lingual UHL and NH on his contralateral ear. The etiology of his hearing loss was unknown, and his middle ear status was normal. He had a duration of deafness of 11 months before unilateral implantation with a CI. Patient S2 was a 51-year-old Caucasian German man also with post-lingual UHL and NH on his contralateral ear. His hearing loss was caused by acoustic trauma; his middle ear status was reported to be normal. Patient S2 had a duration of deafness of 45 months before unilateral CI implantation. Patient S3 was a 48-year-old Caucasian German man with post-lingual UHL and NH on his contralateral ear. He had lost his hearing due to a stapedectomy; he showed a normal middle ear status. Patient S3 was implanted unilaterally with a CI after a duration of deafness of 96 months. Patient S4 was a 57-year-old Caucasian German man with post-lingual UHL and NH on his contralateral ear. His hearing loss was due to a *Borrelia *infection. Patient S4 also showed a normal middle ear status and was implanted with a CI after a duration of hearing loss of 33 months.

Our patients did not wear hearing aids (HAs) before implantation as their hearing loss (HL) was too profound for HAs to provide sufficient acoustic amplification. Since all our patients were members of the German Army, which is obliged by law to provide the best possible compensation for any kind of disability, treatment costs were no issue. Our patients, who all had leading positions within the army, consistently reported a high level of distress often related to feelings that their job was at risk because of their HL and were thus highly motivated for CI treatment. All our patients were thoroughly counseled and signed informed consent before implantation. Authorization was provided by the Germany Army, as our patients were treated in an army hospital in Germany.

All our patients were implanted with the PULSARCI^100 ^implant and a standard electrode of 31.5 mm in length (MED-EL, Innsbruck, Austria). Our patients also received the OPUS 2 speech processor including the FineStructure coding strategy. In all patients, first fitting of the CI was performed approximately four weeks after implantation and included the adjustment of electrical hearing thresholds, the most comfortable stimulation levels as well as frequency allocation and compression characteristics. Our patients received aural rehabilitation therapy for patients with CI in their hearing centers. Bilateral testing was performed once a monosyllable understanding in quiet conditions of 50% correctness was achieved.

**Figure 1:**
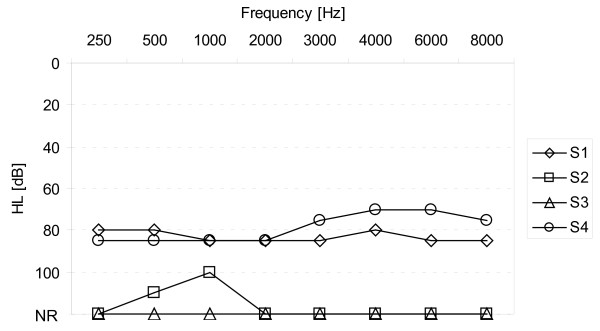
**Individual hearing thresholds of deaf sides before implantation**.

**Figure 2:**
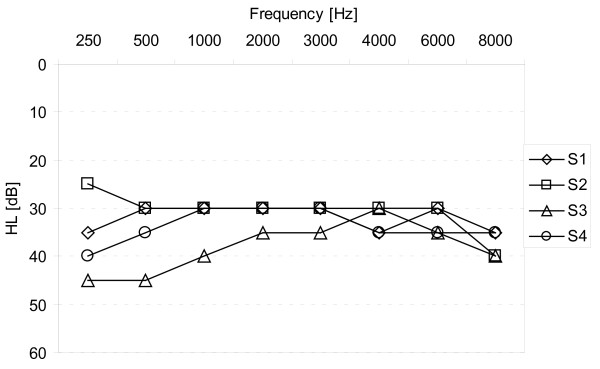
**Individual hearing thresholds in the cochlear implant (CI)-only condition after six months of implant use**. The contralateral ear was plugged and masked with 80 dB broadband noise.

Pure tone audiometry testing was performed in the NH ear, in the hearing impaired ear before CI implantation and, post-operatively, in the CI-only condition six months after first fitting. During testing in the CI-only condition, the contralateral ear was plugged and masked with 80 dB wideband noise. All our patients had NH on the contralateral side with mean hearing thresholds of 5 dB to 30 dB HL across tested frequencies (250 kHz to 8 kHz). Pre-operatively, our patients showed pure tone thresholds averaging between 80 dB and 100 dB in the hearing impaired side (Figure 1). Pure tone audiometry after six months of implant use showed that hearing thresholds were stable in the implanted side and ranged from 25 dB to 45 dB HL (Figure 2).

In the speech tests, our patients were tested with their normal everyday processor settings, which were obtained during clinical device fitting when the implant was first fitted, and our patients subsequently adjusted the loudness to a comfortable level. Tests were performed in an audiometric room with a semicircular loudspeaker set-up. A total of 11 WESTRA audiometry loudspeakers were mounted on a steel ring of 2 m diameter at a height of 1.2 m above the mesh in the frontal horizontal plane from 90° (right) to -90° (left) and with a separation of 18°. Our patients were positioned on an adjustable chair in the center of the semicircle of loudspeakers. All tests, the tested conditions and the time of testing are displayed in Table 1. Test conditions included the acoustic-only (CI side with speech processor turned off and NH side unplugged and unmasked), binaural (NH and CI side) and CI-only (CI side only with contralateral ear plugged and masked with 80 dB wideband noise) conditions.

**Table 1:** Test overview

	S1	S2	S3	S4
Monosyllable test; CI-only*	6	6	6	6
Monosyllable test; acoustic-only and binaural*	22	11	14	12
HSM; acoustic-only and binaural*	22	11	14	12
OLSA; acoustic-only and binaural*	22	11	14	12
Dichotic listening test, CI-only, acoustic-only and binaural*	3	3	3	3
Audiogram of normal hearing ear	Pre-operative	Pre-operative	Pre-operative	Pre-operative
Audiogram of hearing impaired ear (acoustic-only)	Pre-operative	Pre-operative	Pre-operative	Pre-operative
Audiogram of implanted ear (CI-only)*	6	6	6	6

*All time given in months of device use.

CI = cochlear implant; HL = hearing loss; HSM = Hochmair-Schulz-Moser test; OLSA = Oldenburg Sentence Test.

**Figure 3:**
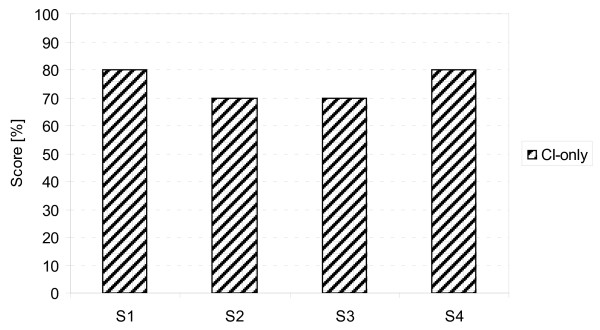
**Freiburg monosyllable test scores in the cochlear implant (CI)-only condition with stimuli presented at 65 dB sound pressure level (SPL) in quiet conditions on the CI side, with the contralateral ear plugged and masked, after six months of implant use**.

**Figure 4:**
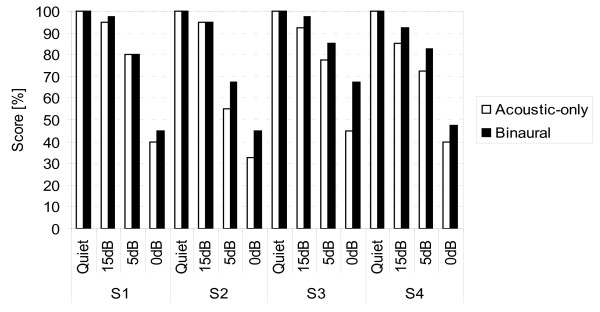
**Freiburg monosyllable test scores in binaural and acoustic-only conditions with stimuli presented at 65 dB sound pressure level (SPL) in quiet conditions and at different signal-to-noise ratios (SNRs) with stimuli (and CCITT noise) presented from the front, with the contralateral ear unplugged and unmasked**.

The Freiburg monosyllable test [11] (20 lists, 20 monosyllable words per list) was presented at a sound pressure level (SPL) of 65 dB SPL. Stimuli were presented from the front in quiet conditions and at a signal-to-noise ratio (SNR) of 15 dB, 5 dB and 0 dB, and speech simulating CCITT (Comite Consultatif International Telegraphique et Telephonique) noise was presented from the front (same loudspeaker). The test was performed in acoustic-only, binaural and CI-only conditions. Monosyllable scores in the CI-only condition are shown in Figure 3. After six months, our patients scored between 70% and 80% (mean: 75%). The individual monosyllable scores for binaural and acoustic-only conditions are shown in Figure 4. Consistent ceiling effects were observed in quiet conditions for both the acoustic-only and binaural conditions. A consistent increase in speech understanding in the binaural condition compared to the acoustic-only condition was found at all three SNR levels: the mean benefit at 15 dB SNR was 3.8 percentage points (pp), at 5 dB SNR 7.5 pp and at 0 dB SNR 11.9 pp (Figure 4).

**Figure 5:**
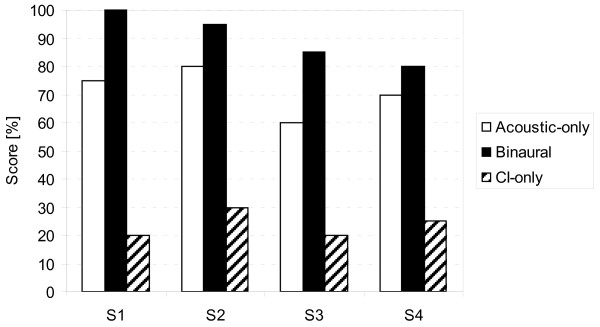
**Individual dichotic two-digit number scores at 80 dB sound pressure level (SPL): acoustic-only, binaural and cochlear implant (CI)-only (contralateral ear plugged and masked) conditions three months after first fitting with signals presented from -90° and 90°**.

The dichotic listening test [12] included 10 number groups of which each contained 10 two-digit number pairs that were presented simultaneously at 80 dB SPL via loudspeakers. The use of loudspeakers deviated from the original test design suggested by Feldmann [12] and was selected to emphasize the impact of the head shadow effect and binaural hearing. This test was performed in acoustic-only, binaural and CI-only conditions. Dichotic speech test results are depicted in Figure 5. Scores ranged from 60% to 80% (mean: 71.3%) acoustic-only, 20% to 30% (mean: 23.8%) with CI-only (with contralateral ear plugged and masked) and 80% to 100% (mean: 90%) in the binaural condition. The mean benefit in the binaural condition compared to the acoustic-only condition was 18.8 pp.

**Figure 6:**
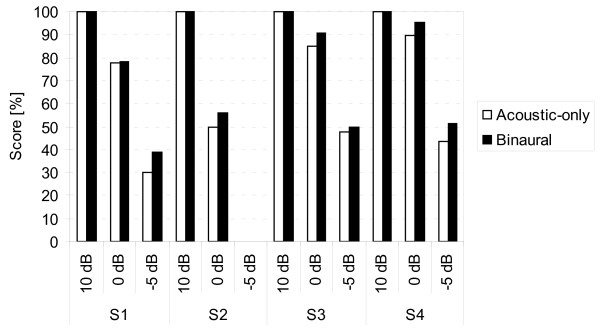
**Individual Hochmair-Schulz-Moser (HSM) sentence tests scores at 65 dB sound pressure level (SPL) at different signal-to-noise ratios (SNRs) (CCITT noise) presented from the front in binaural and acoustic-only conditions, with the contralateral ear unplugged and unmasked**.

The Hochmair-Schulz-Moser (HSM) sentence test [13] (30 lists of 20 sentences three to eight words long, each list consisting of 106 words) was presented at 65 dB SPL with a SNR of 10 dB, 0 dB and -5 dB. The test was performed in acoustic-only and binaural conditions. Individual HSM scores in the binaural and acoustic-only conditions are shown in Figure 6. Ceiling effects were observed at 10 dB SNR. At 0 dB SNR and -5 dB SNR, the HSM scores of all our patients were higher in the binaural condition compared to the acoustic-only condition with a mean increase in speech understanding of 4.6 pp at 0 dB SNR and of 6.3 pp at -5 dB SNR.

**Figure 7:**
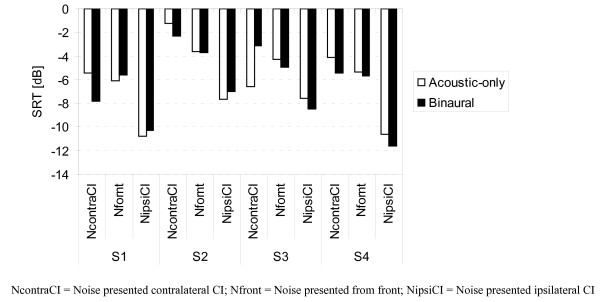
**Mean Oldenburg Sentence Test (OLSA) speech reception thresholds (SRTs) at 60 dB sound pressure level (SPL) noise level and adaptive speech in binaural and acoustic-only conditions, with speech presented from the front and noise presented from front, contralateral cochlear implant (CI) and ipsilateral CI**. NcontraCI = noise presented contralateral CI; Nfront = noise presented from front; NipsiCI = noise presented ipsilateral CI.

The Oldenburg Sentence Test (OLSA) [14] (30 sentences, five words per sentence) was modified by presenting speech from various azimuth conditions, that is, speech was presented either from the front, contralateral CI or ipsilateral CI. The noise was presented from the front and its level was constant (60 dB SPL), whereas the speech level was varied adaptively. For each test run, individual speech reception thresholds (SRTs) were calculated by averaging the signal levels of the last 20 sentences in each list and subtracting the noise level of 60 dB SPL. The test was performed in acoustic-only and binaural conditions. Each condition (listening and azimuth conditions) was tested twice in a randomized order. Individual SRTs measured with the OLSA are shown in Figure 7. Our patients performed best when the noise signal was presented on the CI side (-9.3 dB binaural, -9.2 dB acoustic-only) and worst when the noise was presented on the contralateral side (-4.7 dB binaural, -4.3 dB acoustic-only). With noise presented from the front, our patients performed slightly better than in the contralateral presentation (-5.0 dB with CI, -4.8 dB acoustic-only). Mean differences between listening conditions were less than 0.4 dB for all noise source placements.

## Discussion

Specific testing decisions were taken considering the dichotic listening test, whose design deviated from the original test layout [12] by using loudspeakers instead of headphones. Thus, we could focus not only on the perception of two highly separated sounds but also on head shadow and binaural effects. The positive results of this test suggest that unilaterally implanted patients may benefit from a reduced head shadow, which in this case obstructed speech sources from the hearing ear, as well as from binaural effects known for NH individuals or bilateral CI users and the ability to integrate independent inputs on both sides.

The results of all speech tests demonstrate that patients benefit from binaural hearing when compared to the acoustic-only or CI-only conditions. However, due to the small number of patients tested, conclusions about the statistical significance cannot be drawn.

To obtain a tendency of the subjective perception, we used a visual analog scale (VAS), ranging from 0 (very low) to 10 (very high). Our patients stated a high level of CI acceptance, integration of CI hearing, increased ease of listening especially in noise situations and a regaining of acoustic orientation abilities. After our patients became accustomed to the CI sound, they also rated the quality of the sound signals generated by the CI to be good. No negative interference of NH when using the CI was reported. This can probably be attributed to advancing CI technology having developed modern coding strategies with high frequency resolution and temporal processing. Overall, the subjective ratings of the CI were more positive than results of the objective testing.

It should be emphasized, however, that the VAS was not validated, but shows a tendency of how the CI is perceived. It must furthermore be stated that the subjective results might possibly be influenced by psychological effects, such as our patients' high motivation and expectations towards CI implantation. It might thus be interesting for future studies to focus in greater detail on the subjective benefits of unilateral CIs in particular, as well as on possible psychological effects influencing the subjective perception.

This contrast between objective and subjective benefits might also be due to the fact that the speech tests are designed for bilateral HL and not for NH on the contralateral side. The results in the acoustic-only condition might be dominated by the NH ear, thus decreasing the measurable difference between acoustic-only and binaural test results. A further explanation might be that all our patients reported improved sound localization abilities, which, however, cannot be fully reflected in speech tests, even if two separately placed speech sources are used. It would be an attractive aspect for future unilateral CI studies to include localization tests and to investigate if subjective and objective results continue to show different levels of improvement. Furthermore, future studies should include a greater subject population and adapted speech tests focusing on unilateral hearing so that statistical significance can be demonstrated.

Our patients in the present study had profound UHL for which HAs would not have rendered sufficient acoustic amplification. Since all our patients were members of the German army, treatment costs were no issue; however, generally, cost effectiveness plays an important role in health care structures. Regardless, based on the results of studies by Bond *et al. *[15], for example, the decision of unilateral cochlear implantation should not be influenced or even restrained by cost-related arguments.

## Conclusions

Our patients showed a small but important benefit from unilateral cochlear implantation in speech recognition in noise conditions. The subjective benefits suggest a high degree of integration of the artificial auditory input through the CI. Restored sound localization and a regained spatial awareness were also reported and might be of interest in future research.

## Consent

Written informed consent was obtained from the patients for publication of this case report and any accompanying images. A copy of the written consent is available for review by the Editor-in-Chief of this journal.

## Competing interests

The authors declare that they have no competing interests.

## Authors' contributions

All authors contributed to the manuscript and reviewed, edited and approved the final report.
